# Effects of Normal Synovial Fluid and Interferon Gamma on Chondrogenic Capability and Immunomodulatory Potential Respectively on Equine Mesenchymal Stem Cells

**DOI:** 10.3390/ijms22126391

**Published:** 2021-06-15

**Authors:** Mohammed Zayed, Steve Adair, Madhu Dhar

**Affiliations:** 1 Department of Large Animal Clinical Sciences, College of Veterinary Medicine, University of Tennessee, Knoxville, TN 37996, USA; mzayed2@vet.svu.edu.eg (M.Z.); sadair@utk.edu (S.A.); 2 Department of Surgery, College of Veterinary Medicine, South Valley University, Qena 83523, Egypt

**Keywords:** mesenchymal stem cells, synovial fluid, chondrocyte differentiation, immunomodulatory, IFN-γ

## Abstract

Synovial fluid contains cytokines, growth factors and resident mesenchymal stem cells (MSCs). The present study aimed to (1) determine the effects of autologous and allogeneic synovial fluid on viability, proliferation and chondrogenesis of equine bone marrow MSCs (BMMSCs) and (2) compare the immunomodulatory properties of equine synovial fluid MSCs (SFMSCs) and BMMSCs after stimulation with interferon gamma (INF-γ). To meet the first aim of the study, the proliferation and viability of MSCs were evaluated by MTS and calcein AM staining assays. To induce chondrogenesis, MSCs were cultured in a medium containing TGF-β1 or different concentrations of synovial fluid. To meet the second aim, SFMSCs and BMMSCs were stimulated with IFN-γ. The concentration of indoleamine-2,3-dioxygenase (IDO) and nitric oxide (NO) were examined. Our results show that MSCs cultured in autologous or allogeneic synovial fluid could maintain proliferation and viability activities. Synovial fluid affected chondrocyte differentiation significantly, as indicated by increased glycosaminoglycan contents, compared to the chondrogenic medium containing 5 ng/mL TGF-β1. After culturing with IFN-γ, the conditioned media of both BMMSCs and SFMSCs showed increased concentrations of IDO, but not NO. Stimulating MSCs with synovial fluid or IFN-γ could enhance chondrogenesis and anti-inflammatory activity, respectively, suggesting that the joint environment is suitable for chondrogenesis.

## 1. Introduction

The native minimal regeneration of the articular cartilage is a primary challenge for cartilage healing. The shortage of successful repair also contributes to the prevalent degeneration of the joint related to osteoarthritis (OA). The current approaches used to regenerate the articular cartilage commonly result in the formation of fibrocartilage tissue which is of low-grade quality compared to the intrinsic hyaline articular cartilage [1,2]. Recently, decellularization of cartilage tissue with post-decellularization methods and, finally, recellularization with or without stem cells appeared to be a promising approach for effective repair of damaged cartilage tissue [3]. Cell-based therapies are progressively used as a potential treatment for cartilage regeneration. Chondrogenic differentiation of mesenchymal stem/stromal cells (MSCs) has potential for clinical applications. Therefore, MSCs are considered a promising approach to regenerate damaged cartilage with engineered tissue [4,5]. Autologous chondrocyte implantation is used to treat articular cartilage defects. However, with its high cost, together with the regeneration of fibrous tissue rather than hyaline cartilage, this method does have its limitations [6,7].

Intra-articular implantation of chondrogenic-generated stem cells (MSCs induced in vitro towards chondrogenic lineage) to regenerate damaged cartilage has been used in previous studies [8]. Based on the results of previous studies, the administration of allogeneic chondrogenic-generated MSCs into the joint has proven to be an effective treatment of degenerative joint disease in horses [9,10]. However, long-term in vitro culturing can lead to undesirable changes in chondrocyte phenotype [11], such as chondrocyte dedifferentiation, cell death and cell leakage [12]. Moreover, an appropriate new environment for the development of highly efficient MSCs with chondrogenic differentiation potential within a relatively short time is necessary.

Synovial fluid has a key role in nourishing articular cartilage, lubricating the articular joint and absorbing shock. In addition, synovial fluid contains growth factors, such as the transforming growth factor (TGF-β1) [13]. In a study conducted by Chen et al., researchers co-cultured bone marrow-derived MSCs (BMMSCs) from sheep in presence of synovial fluid. Results demonstrated that MSCs were capable of differentiating into chondrocytes and did express chondrogenesis markers [14]. In fact, some reports prove that the synovial fluid might stimulate chondrocytes differentiation [15,16]. Data also indicate that exposing MSCs to synovial fluid might alter their chondrogenesis phenotype and, as a result, the response of MSCs to synovial fluid is not consistent. In equines, autologous and allogeneic MSCs can be used for the treatment of musculoskeletal disorders [17], modifying inflammatory processes [18] and have demonstrated no adverse immunological reactions to intra-articular injection [19]. Therefore, the first aim of our study is designed to determine the survival rate and chondrogenic effects of autologous and allogenic synovial fluid on equine BMMSCs.

It has been proven that synovial fluid from normal and osteoarthritic joints has a resident MSC population [20]. Advanced osteoarthritis is associated with a numerical increase in MSCs characterized by a stem cells marker and a potential of trilineage differentiation [21,22]. MSCs have various mechanisms in regenerative medicine for clinical applications. One of these mechanisms is their anti-inflammatory and immunosuppressive properties. Under homeostatic conditions, the immunomodulatory ability of MSCs is considered hypoimmunogenic, but, under inflammatory conditions, it is activated by various inflammatory cytokines [23]. Interferon-gamma (IFN-γ) plays a key role in the stimulation of immunomodulatory activities of MSCs [24,25]. Interferon-γ is a cytokine, which is largely involved in the immune response of cartilage diseases [26,27]. It is produced mainly by natural killer cells, activated CD4+ Th1 cells and cytotoxic CD8+ cytotoxic T cells [28]. Production of indoleamine-2,3-dioxygenase (IDO) is one of the major immunosuppression mechanisms of equine MSCs [29]. IDO is implicated in L-tryptophan catabolism and leads to the accumulation of kynurenine. Kynurenine is essential in inhibiting the proliferation and activation of T cells and inhibiting natural killer cell proliferation and activity [30]. In addition, the production of nitric oxide (NO) is able to suppress or modulate immune responses [31]. We have previously characterized the equine bone marrow and synovial fluid derived MSCs with respect to their in vitro proliferation, trilineage differentiation and immunophenotyping. Using these assays, we showed that both cell types adhered to the tissue culture substrate, proliferated, had a potential to undergo trilineage differentiation, exhibited >70% expression of CD29, CD44 and CD90 and lacked the expression of CD34 and MHC-II [20,32]. These in vitro studies confirmed the identity and “stemness” of equine MSCs isolated from bone marrow and synovial fluid. While equine SFMSCs have been previously characterized in terms of their growth, multipotency and CD profile, the immunological and immunomodulatory properties under inflammatory stimulation have not been thoroughly defined. Thus, the second aim of our study is to investigate the immunomodulatory properties of equine SFMSCs and BMMSCs under IFN-γ stimulation.

## 2. Results

### 2.1. Morphology and Colony Forming Unit of MSCs

**Figure 1:**
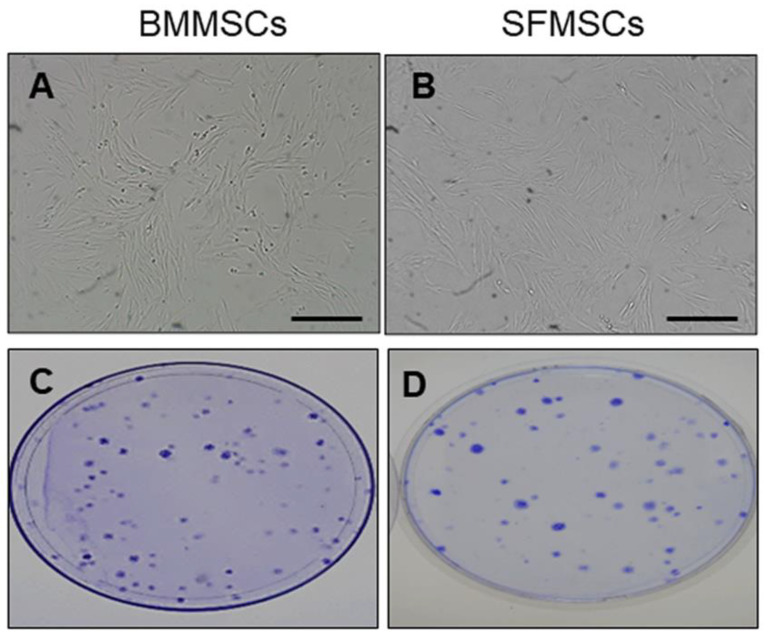
Morphology and colony forming unit (CFU) of MSCs from bone marrow and synovial fluid during cell culture. Representative microscopic images of MSCs from bone marrow (**A**) and synovial fluid (**B**) with approximately 80% confluence at 100× magnification. (**C**,**D**) CFU assay of bone marrow and synovial fluid derived-MSCs, respectively, cultivated as 100 cells per 100  cm^2^ culture dish for 7 days. The colonies stained with 0.5% crystal violet.

In the current study, we evaluated the morphology of both cell populations of MSCs isolated from a subset of horses (*n* = 3) used in previous studies. In this study we reconfirmed the identity of the previously characterized MSCs using cell morphology and colony forming unit assay. Equine BMMSCs and SFMSCs showed a large cluster of stellate cells and growth in a fibroblast-like phenotype (Figure 1A,B). Further cultivation revealed proliferation and multiplication while maintaining their morphology with homogeneous appearance. Approximately 10^2^ cells of both cell sources were able to form adherent colonies (Figure 1C,D), a characteristic feature of stromal stem-cell populations.

### 2.2. Effect of Synovial Fluid on Proliferation and Cell Viability of MSCs

**Figure 2:**
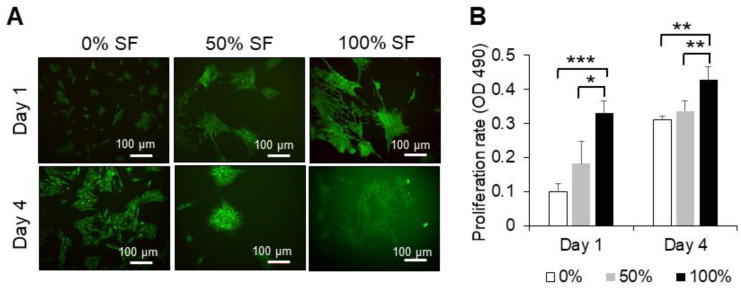
Proliferation rate and calcein staining for viability of BMMSCs co-cultured with autologous synovial fluid. (**A**) Representative BMMSCs after exposure to different concentrations of autologous synovial fluid at days 1 and 4. MSCs were loaded with calcein AM (stains live cells green) and propidium iodide (stains dead cells red). (**B**) Proliferation was evaluated by MTS assay; the absorbance was measured at 490 nm. * *p* < 0.05, ** *p* < 0.01 and *** *p* < 0.001. Quantitative data are expressed as mean ± SD (*n* = 3) and were analyzed using one-way ANOVA, with Tukey’s comparison test as a posttest. *p* < 0.05 was considered statistically significant. One representative experiment is presented from three different experiments using cells from different donors (*n* = 3), each performed in triplicate. Scale bars = 100 µm.

**Figure 3:**
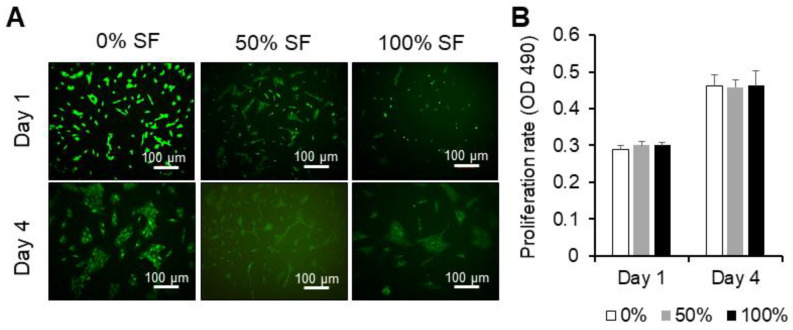
Proliferation rate and calcein staining for viability of BMMSCs co-cultured with allogeneic synovial fluid. (**A**) Representative BMMSCs after exposure to different concentrations of allogeneic synovial fluid at days 1 and 4. MSCs were loaded with calcein AM (stains live cells green) and propidium iodide (stains dead cells red). (**B**) Proliferation was evaluated by MTS assay; the absorbance was measured at 490 nm. Quantitative data are expressed as mean ± SD (*n* = 3) and were analyzed using one-way ANOVA, with Tukey’s comparison test as a posttest. *p* < 0.05 was considered statistically significant. One representative experiment is presented from three different experiments using cells from different donors (*n* = 3), each performed in triplicate. Scale bars = 100 µm.

Proliferation rate and cellular viability of BMMSCs cultured in a regular medium, 50% of autologous and allogenic synovial fluid and 100% of autologous and allogenic synovial fluid, were assessed by MTS assay and calcein AM, respectively, at days 1 and 4. When co-cultured with synovial fluid, MSCs changed their shapes, appeared to aggregate into grid-like groups and then clumped (Figure 2A and Figure 3A).

Autologous synovial fluid of 100% concentration demonstrated a significant difference in the proliferation rate, compared to 50% of synovial fluid and the regular medium at day 1 (*p* < 0.05 and *p* < 0.001, respectively) and at day 4 (*p* < 0.01 and *p* < 0.01, respectively). However, different concentrations of allogeneic synovial fluid could only maintain proliferation and did not show any difference compared to the regular medium containing DMEM-F12, 10% FBS and 1% penicillin/streptomycin (Figure 2B and Figure 3B). Results indicate that the different concentrations of autologous and allogeneic synovial fluid could maintain the viability of BMMSCs.

### 2.3. Synovial Fluid as Chondrogenesis Inducing Factor

**Figure 4:**
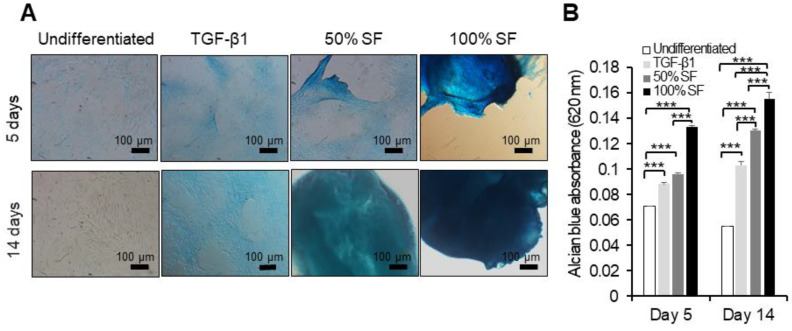
Evaluation of chondrogenic differentiation in the presence of autologous synovial fluid. BMMSCs were cultivated as monolayer in the presence of 50% and 100% of autologous synovial fluid or chondrogenic differentiation medium containing TGF-β1 for up to 14 days. The MSCs were stained using Alcian blue. (**A**) Photomicrographs of MSCs at days 5 and 14 show cartilage glycosaminoglycans (blue) indicative of chondrogenic differentiation. (**B**) Total glycosaminoglycan (GAG) production in the monolayer at different time points. *** *p* < 0.001. Quantitative data are expressed as mean ± SD (*n* = 3) and were analyzed using one-way ANOVA, with Tukey’s comparison test as a posttest. *p* < 0.05 was considered statistically significant. One representative experiment is presented from three different experiments using cells from different donors (*n* = 3), each performed in triplicate.

**Figure 5:**
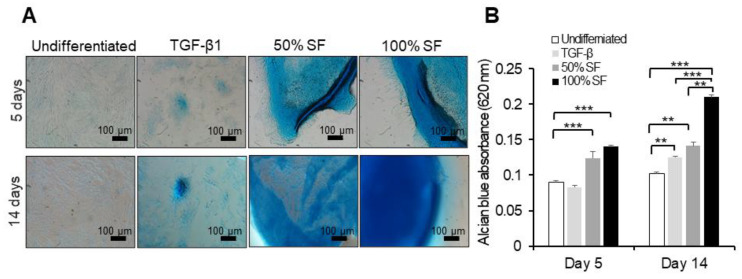
Evaluation of chondrogenic differentiation in the presence of allogeneic synovial fluid. BMMSCs were cultivated as monolayer in the presence of 50% and 100% of allogeneic synovial fluid or chondrogenic differentiation medium containing TGF-β1 for up to 14 days. The MSCs were stained using Alcian blue. (**A**) Photomicrographs of MSCs at days 5 and 14 show cartilage glycosaminoglycans (blue) indicative for chondrogenic differentiation. (**B**) Total glycosaminoglycan (GAG) production in the monolayer for different time points. ** *p* < 0.01, *** *p* < 0.001. Quantitative data are expressed as mean ± SD (*n* = 3) and were analyzed using one-way ANOVA, with Tukey’s comparison test as a posttest. *p* < 0.05 was considered statistically significant. One representative experiment is presented from three different experiments using cells from different donors (*n* = 3), each performed in triplicate.

To examine the influence of different concentrations of autologous and allogenic synovial fluid on chondrogenic differentiation, equine BMMSCs were cultured up to 14 days in monolayer cultures. The potential of MSCs to differentiate along the chondrogenic lineage was illustrated by the staining of extracellular cartilage matrix proteoglycans with Alcian blue (Figure 4A and Figure 5A).

Our results demonstrate that both autologous and allogenic synovial fluid induced early chondrogenesis starting at day 5, compared to the traditional chondrogenic medium, which induced chondrogenesis after 14 days, as indicated by GAG content measured by Alcian blue absorbance (Figure 4B and Figure 5B). Undifferentiated cells did not show differentiation, i.e., without stimulation with synovial fluid or TGF-β no differentiation was found.

Stimulation of BMMSCs with autologous synovial fluid or regular chondrogenic medium showed a significant increase in their GAG content compared to unstimulated cells (*p* < 0.001) at days 5 and 14. Adding 100% concentration of synovial fluid exhibited significantly higher GAG content in BMMSCs compared to 50% (*p* < 0.001) at days 5 and 14. Moreover, the 50% and 100% concentrations of synovial fluid induced a high amount of GAG compared to the regular chondrogenic medium (*p* < 0.001) at day 14 (Figure 4A,B).

Allogeneic synovial fluid (50% and 100% concentrations) showed a significant increase in chondrogenesis compared to undifferentiated cells (*p* < 0.001) at day 5, but not compared to the regular chondrogenic medium. Moreover, the 50% and 100% concentrations of synovial fluid and regular chondrogenic medium induced a high amount of GAG compared to undifferentiated MSCs at day 14. The 100% concentration of allogeneic synovial fluid showed higher GAG contents than the 50% concentration and regular chondrogenic medium (*p* < 0.001) at day 14.

### 2.4. Immunomodulatory Properties of BMMSCs and SFMSCs under IFN-γ Stimulation

**Figure 6:**
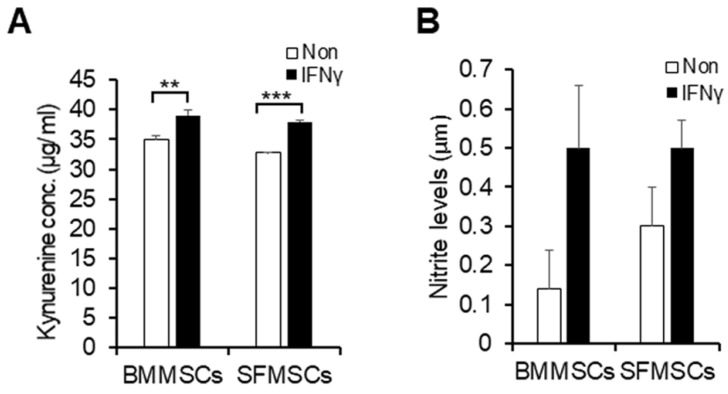
Indoleamine-2,3-dioxygenase (IDO) and nitric oxide (NO) expressions in the conditioned medium stimulated with interferon-gamma (IFN-γ). (**A**) Kynurenine assay, the functional IDO activity was evaluated by measuring the content of kynurenine in the conditioned media of BMMSCs and SFMSCs stimulated with IFN-γ (100 ng/mL) for 48 h. ** *p* < 0.01, *** *p* < 0.001. (**B**) Concentration of nitrite following stimulation with IFN-γ for 48 h. All values represent mean ± SD (*n* = 3). Statistical analysis was carried out using the Student’s t-test with *p* < 0.05 as significant. Experiments were performed on cells from three different donors (*n*  =  3), each in triplicate.

After a 48 h treatment with 100 ng/mL of IFN-γ, the measured concentration of IDO in the conditioned medium, assessed via the production of kynurenine, showed that BMMSCs and SFMSCs exhibited significant secretion of IDO (*p* < 0.01 and *p* < 0.001, respectively) compared to their respective controls (non-stimulated MSCs) (Figure 6A). In monolayer culture, the MSCs produced a high level of kynurenine without IFN-γ stimulation and IFN-γ stimulation augments only about 15%. Moreover, IFN-γ-induced nitrite is not significantly different compared to unstimulated cells (Figure 6B).

## 3. Discussion

Because of the inferior self-repair ability of cartilage, cartilage engineering is a new treatment approach to treat osteoarthritic or traumatic cartilage defects. Based on their chondrogenic and immunosuppressive capacity, a cell-based therapeutic strategy using MSCs has been developed for cartilage regeneration. Because of the prevalence of intra-articular injections of BMMSCs in equine regenerative medicine, we decided to use these cells in our study as a comparison with SFMSCs [33]. Furthermore, in order to improve the feasibility of using these in the clinic, we chose to evaluate MSCs derived from the same recipient donor (autologous) or another donor (allogeneic) [34]. Autologous MSCs have a less immunologic reaction; however, using these MSCs has proven to be costly and time-consuming in vitro [17]. While allogeneic MSCs have the advantage to offer an “off-the-shelf” therapy, their safety must be well established before clinical use. Implementing the direct injection of MSCs into the joint to stimulate cartilage repair and relieve clinical signs of osteoarthritis is common [33,35]. The survival, and whether the joint environment maintains the chondrogenesis of injected MSCs, however, has not been thoroughly investigated. In the current study, we co-cultured equine BMMSCs with synovial fluid, the main component of the natural environment of the joint. Furthermore, the effects of autologous and allogeneic synovial fluid on the survival and chondrogenic differentiation of BMMSCs were evaluated. Moreover, the immunomodulatory activity of MSCs derived from synovial fluid and bone marrow was further examined.

An earlier study demonstrated a significant increase in the cellularity of chondrocytes by supplement with synovial fluid, as indicated by DNA content [16]. Additionally, it has been shown that synovial fluid from rheumatoid patients maintained adipose tissue-derived MSCs proliferative properties [36]. Consistent with earlier studies, our results show that autologous synovial fluid at a concentration of 100% could significantly increase the proliferation rate of BMMSCs up to day 4, compared to concentrations of 0% and 50%. Allogeneic synovial fluid could maintain BMMSCs proliferation, but the impact was not significant, compared to concentrations of 0% and 50%. There was no difference between the effect of 100% concentration of autologous and allogeneic synovial fluid. Furthermore, both autologous and allogeneic synovial fluid maintain the viability of BMMSCs with different concentrations. These data suggest that both autologous and allogeneic synovial fluid support the survival of BMMSCs.

Many reports show chondrogenic differentiation of MSCs in vitro depending on the use of chondrogenic factors. The growth factors of the TGF-β superfamily prompt chondrogenesis of MSCs in vitro [37]. However, in addition to the particular markers of cartilage, TGF-β also produces an in vitro expression of type X collagen, a hypertrophic chondrocyte and an osteoarthritis marker [11]. The chondrocytes develop and form cartilage in the natural environment of the normal joint cavity. Synovial fluid is the main constituent in this environment and acts as an important factor to optimize the conditions to differentiate MSCs toward cartilage. Moreover, synovial fluid is an enriched source that contains different factors, mainly glycosaminoglycans. Hyaluronic acid (HA) and chondroitin sulphate are the major glycosaminoglycans that have been found in synovial fluid in different species and, hence, the effect of HA on the chondrocytes and the MSCs implanted should be considered [38]. Multiple in vitro studies have shown the effect of hyaluronic acid on chondrocytes [16,39]. Hyaluronic acid increases cell proliferation and chondrogenesis, enhances chondrocyte survival, improves mitochondrial function, increases the production of CS by chondrocytes and, finally, it suppresses matrix metalloproteinases [40,41]. Hence, it is important not to disregard the interaction between HA and MSCs in therapies mediated by either the BMMSCs or SFMSCs. Thus, it is crucial to examine the effect of synovial fluid, both autologous and allogeneic, on the chondrogenic differentiation of MSCs. To do that, we co-cultured BMMSCs in presence of media containing 50% and 100% of synovial fluid. The results of our study demonstrate that both autologous and allogeneic synovial fluid can induce the differentiation of MSCs to chondrocytes in vitro even more than the regular chondrogenic medium that contains TGF-β. The results of our study are in agreement with some earlier studies that showed that synovial fluid increases chondrogenesis in vitro [15,16]. Our results also support the study in which BMMSCs were injected into the normal joint cavity and expressed type II collagen and synthesized sulfated proteoglycans in the sheep model [14]. One reason that synovial fluid potentially induces chondrogenesis is due to the fact that it contains many bioactive factors that may enhance the chondrogenic differentiation of BMMSCs [13,42]. Hyaluronic acid is a factor that was reported to induce MSC chondrogenesis and is used to treat osteoarthritis in horses [43].

In veterinary practice, MSCs have many advantages for clinical applications, such as differentiating into multiple tissue types. In addition, MSCs have a potent anti-inflammatory/immunomodulatory property to treat inflammation [44,45]. It has been proven that equine SFMSCs stimulate regeneration of full-thickness articular cartilage defect in rats [46]. To investigate the immunomodulatory effect of SFMSCs and BMMSCs, we stimulated MSCs with IFN-γ. The properties of MSCs are not always immunomodulatory, but they may have a different immunomodulatory outline, controlled by the local environment. For instance, under the inflammatory condition, where there are elevated levels of pro-inflammatory cytokines (IFN-γ, TNF-α and IL-1β), MSCs were stimulated and demonstrated an immunosuppressive phenotype. Preconditioning of equine BMMSCs with IFN-γ increased the immunosuppressive properties of MSCs, according to previous studies [45,47]. Moreover, upon IFN-γ stimulation, MSCs increased IDO and secreted essential anti-inflammatory factors, such as TGF-β, HGF, PGE2 [48,49]. The immunosuppressive ability of MSCs is also depicted by the increase of NO products, which plays a main role in inhibiting T-cell proliferation [50]. Our results are consistent with previous studies and further prove that stimulation by IFN-γ enhances the immunosuppressive properties of MSCs by upregulating IDO production. However, there is no significant difference in NO production between the stimulated and non-stimulated MSCs, which could depend on the low concentration of IFN-γ [51,52]. In agreement with our in vitro results, Williams and his colleagues demonstrated that allogeneic umbilical MSCs decreased the inflammation on joints induced by LPS in an in vivo equine study [53]. Even though our in vitro data complement or are supported by both in vitro and in vivo observations reported in literature, further studies to confirm the in vivo applicability are needed before this strategy can be translated into the clinic.

## 4. Materials and Methods

All animal procedures were carried out in accordance with the protocols approved by the University of Tennessee Institutional Animal Care and Use Committee (protocol number 1953, 27 August 2014–2017). Six healthy horses (aged 8–12 years) were enrolled in this study.

### 4.1. Isolation and Culture of MSCs

Isolation, characterization and expansion of BMMSCs were carried out as described previously [54] from 3 previously sampled horses [20]. To summarize, the mononuclear fraction of the BM was seeded in a growth medium containing Dulbecco’s modified Eagle’s medium/Ham’s F-12 (DMEM-F12; Fisher Scientific, Hampton, NH, USA), 10% fetal bovine serum (FBS, Merck Animal Health, Summit, NJ, USA) and 1% penicillin/streptomycin (Fisher Scientific, Hampton, NH, USA). After incubation at 37 °C in 5% CO_2_, adherent cells were harvested at 80% confluency with 0.25% trypsin-EDTA (Fisher Scientific, Hampton, NH, USA).

Collection of synovial fluid from normal joints and isolation of SFMSCs were performed according to our previous studies [20,32]. To collect the synovial fluid (SF), animals were tranquilized with detomidine (Pfizer Animal Health, Parsippany, NJ, USA) IV and 2–5 mL of SF was collected under a septic condition from each of the radiocarpal, intercarpal and tarsocrural joints. The samples of SF were first checked by cytological analysis to be normal and then pooled (Table 1). One milliliter of SF was diluted with 9 mL of regular growth medium, i.e., DMEM-F12 supplemented with 10% FBS and 1% penicillin/streptomycin, seeded into 75 cm^2^ vented tissue culture flasks and incubated at 37 °C in 5% CO_2_ and 95% relative humidity. This DMEM-F12 medium is the regular growth medium. Adherent cells were harvested with 0.25% trypsin-EDTA for 1 min at 37 °C and cryopreserved at 2nd–4th passages of culture for the in vitro studies.

The colony forming unit assay was conducted with slight modification, according to traditional methods [20]. Briefly, BMMSCs and SFMSCs (P1, 1 × 10^2^) were seeded on 10 cm^2^ diameter dishes (Thermo Scientific, Waltham, MA, USA) and cultured using DMEM-F12 regular growth medium. When clusters of colonies were formed, at day 7, cells were fixed and stained with 0.5% crystal violet (Sigma-Aldrich, Saint louis, MO, USA).

### 4.2. Cell Proliferation and Viability

Proliferation of expanded BMMSCs (P2-P3, 2 × 10^4^ cells) was evaluated in the presence of autologous and/or allogenic synovial fluid with varying concentrations (0%, 50%, or 100%) at days 1 and 4 using the 3-(4,5-dimethylthiazol-2-yl)-5-(3-carboxymethoxyphenyl)-2-(4-sulfophenyl)-2H-tetrazolium (MTS) assay (Promega, Madison, WI, USA) in 24-well plates. For the assay, 100 µL of the MTS reagent was added to each well, which contained cells in 500 µL of DMEM-F12, regular growth medium, and then incubated for 3 h at 37 °C/5% CO_2_. Regular growth media without cells served as controls. Optical densities were measured on a microplate reader at 490 nm (BioTek, Winooski, VT, USA).

Cell adhesion and viability were also assessed microscopically after days 1 and 4 by means of calcein AM (Invitrogen, Carlsbad, CA, USA) and propidium iodide (Invitrogen, USA) staining. MSCs were seeded at a density of 2.0 × 10^4^ cells per well in a 24-well plate. Cells were stained as per the manufacturer’s protocols and consequently visualized using a Zeiss Axiovert 40 C microscope (Carl Zeiss MicroImaging Inc., Thornwood, NY, USA).

### 4.3. Chondrocyte Differentiation of Equine BMMSCs

These experiments were carried out using optimized conditions as described earlier [32]. BMMSCs were seeded at a density of 2.0 × 10^4^ cells per well in 24 well plates (Thermo Scientific) containing DMEM-F12 regular growth medium. Cells were allowed to adhere to the tissue culture surface. At 70–80% confluency, the medium was replaced with either chondrogenic differentiation medium, or media containing either 50% or 100% of autologous and allogenic synovial fluid. The chondrogenic differentiation medium, i.e., regular medium supplemented with 100 nmol/L dexamethasone, 0.25 mmol/L ascorbic acid (Sigma-Aldrich, Saint louis, MO, USA) and 5 ng/mL transforming growth factor beta (TGF)-β1 (R&D Systems, Minneapolis, MN, USA), was used as positive control. BMMSCs without treatment were kept as negative control. The chondrogenic medium and/or synovial fluid supplementations were changed every 3 days for a 14-day culture period. At days 5 and 14, cells were fixed with 4% paraformaldehyde for 10 min at room temperature (RT) and stained with 0.2% Alcian blue (Sigma-Aldrich, Saint louis, MO, USA) for 1 h at RT. Photomicrographs were examined with the NIS-Elements imaging software (Nikon Instruments, Winooski, VT, USA). A total of 500 μL of 6 M guanidine/HCl (Fisher Scientific, Waltham, MA, USA) was added to the stained cells overnight at RT with shaking to extract Alcian blue. The optical density of the extracted Alcian blue was measured on a microplate reader (BioTek, Winooski, VT, USA) at 620 nm and the values were used as a measure of the GAG content.

### 4.4. Interferon-γ Stimulation

BMMSCs and SFMSCs (P2–P4) were seeded at a density of 1 × 10^5^ cells per well onto a 24-well plate for 48 h to adhere. Cells were then stimulated with IFN-γ (100 ng/mL, PeproTech, Cranbury, NJ, USA) [55] for 48 h. The immunogenic activity of SFMSCs and BMMSCs was investigated by measuring the concentration of L-kynurenine and nitrites in the conditioned medium. Non-stimulated cells cultivated in regular growth medium were used as control for comparison.

### 4.5. Kynurenine Assay

The biological activity of IDO was assessed by measuring the level of the tryptophan degradation resulting in L-kynurenine accumulation in the conditioned medium of stimulated and non-stimulated MSCs after 48 h, according to the previous study, with slight modification [56]. Briefly, the conditioned medium and 30% trichloroacetic acid (Sigma-Aldrich) were mixed at a ratio of 2:1 in a 24-well plate. After 30 min incubation at 50 °C, the plate was centrifuged for 5 min at 1200 rpm. Equal volume of the supernatant and Ehrlich’s reagent (200 µg 4—dimethylaminobenzaldehyde (Sigma-Aldrich) in 10 mL glacial acetic acid) was incubated in the dark at RT for 10 min. A microplate reader reading at 490 nm was used to quantify the kyneurine concentration. According to a standard curve of known concentrations of kynurenine, the concentration of kynurenine in the conditioned medium was calculated.

### 4.6. Griess Assay

Nitric oxide production was verified by measuring its stable end product, nitrite, using a Griess reagent (Promega Corporation, Madison, WI, USA) in the conditioned medium of stimulated and non-stimulated MSCs after 48 h of treatment, according to manufacturer’s protocol. For the assay, 50 μL of the conditioned medium was added to a 96-well plate, followed by 50 μL of sulphanilamide and 50 μL N-1-napthylethylenediamine dihydrochloride (NED). Absorbance at 540 nm was measured by a microplate reader and nitrite concentrations were calculated using a standard nitrite curve.

### 4.7. Statistical Analysis

All the results are expressed as the means ± standard deviation. The data were analyzed statistically using the Student’s t-test or one-way analysis of variance (ANOVA), with Tukey’s comparison test as a posttest, using SPSS 25.0 (IBM, Armonk, NY, USA).

## 5. Conclusions

While we did not recognize the definitive factors, we concluded that synovial fluid is a proper substrate to differentiate MSCs into chondrocytes and proved the concept that the application of MSCs in the joint environment is suitable. SFMSCs demonstrated immunosuppressive properties under inflammatory conditions, which is another hallmark for cartilage regeneration and supports the use of SF as a delivery vehicle for MSCs. Further investigations, however, are required to examine the hyaline cartilage markers (both at the mRNA and at the protein levels) in the differentiated MSCs supplemented with synovial fluid. These studies will help understand the signaling mechanisms that are triggered by SF. Moreover, further in vivo studies to transplant the aggregate differentiated BMMSCs, in presence of synovial fluid, into cartilage defects will be critical for future clinical setting.

## Figures and Tables

**Table 1:** Results of examination of synovial fluid from normal horses.

Determinations and Cytological Values	No. of Synovial Fluid Samples	Results
Total protein (g/dL)	6	2.1 ± 0.1
Total nucleated cell count/uL	6	716 ± 291
Monocytes (%)	6	35.3 ± 11
Neutrophils (%)	6	2.8 ± 1.3
Lymphocytes (%)	6	24 ± 8.9
